# The Burden of Diarrheal Diseases among Children under Five Years of Age in Arba Minch District, Southern Ethiopia, and Associated Risk Factors: A Cross-Sectional Study

**DOI:** 10.1155/2014/654901

**Published:** 2014-11-18

**Authors:** Shikur Mohammed, Dessalegn Tamiru

**Affiliations:** Department of Public Health, Arba Minch University, P.O. Box 25121, Arba Minch, Ethiopia

## Abstract

*Introduction*. In Ethiopia diarrhea is the second cause for clinical presentation among under five-year child population next to pneumonia and it is also more common in rural than in urban areas. *Methods*. A community based cross-sectional study was conducted in Arba Minch District. Data were collected using structured questionnaire by trained data collectors. To identify predictors of diarrhea the negative binomial regression model was used to predict and control the effect of confounders. *Results*. The prevalence of diarrhea among under-five children was 30.5%. This study showed children whose mothers did not attend any formal education were 89% more likely to develop diarrhea (APR = 1.89, [95% CI: 1.35, 2.53]) compared to their counterparts. Similarly, children's being in age category 6-23 months (APR = 2.78 [95% CI: 1.72, 4.55]) and mothers' poor hand washing practice (APR = 2.33 [95% CI: 1.80, 4.15]) were found predictors of diarrhea. The study also showed that, out of 180 mothers whose child had got diarrhea, about 31% of mothers could not give anything to manage the diarrhea. *Conclusions*. In this study the prevalence of diarrhea was high which was significantly associated with maternal education level, age of the child, and personal hygiene practices. Therefore, women's education level of at least primary school and enhancing community based behavioral change communications using multiple channels (radio) and community health workers are recommended to reduce the occurrence and consequences of childhood diarrhea in the study area.

## 1. Introduction

Diarrheal diseases are amongst the most frequent childhood illnesses and leading cause of preventable death, especially among children under five in developing countries. Acute diarrheal diseases are one of the main problems affecting children in the world, reducing their well-being and creating considerable demand for health services [1]. Previous studies showed that diarrheal episode causes the death of 700,000 under-five children. Young children are especially vulnerable to diarrheal disease and the majority of deaths related to diarrhea took place in Africa and South Asia. This is unfortunate since the condition can be easily treated with oral rehydration therapy [1–3].

Diarrhea is defined as a child with loose or watery stool for three or more times during a 24-hour period [1, 4]. The frequency and severity of diarrhea are aggravated by lack of access to sufficient clean water and sanitary disposal of human waste, inadequate feeding practices and hand washing, poor housing conditions, and lack of access to adequate and affordable health care [4]. The etiology of diarrhea must be understood to accelerate additional preventive measures. Optimal infant and young child feeding practices could prevent more than 10 percent of deaths from diarrhea and acute respiratory infections [1, 4]. Better hygiene practices, particularly hand washing with soap and the safe disposal of excreta, could reduce the incidence of diarrhea by 35 percent [2–6].

Studies have shown that, in developing countries like Ethiopia, the occurrence of diarrheal disease among under-five children is complex and the relative contribution of each factor varies as a function of interaction between socioeconomic, environmental, and behavioral variables. In many developing countries sociodemographic characteristics like maternal and child age and availability sanitary facilities, hygienic practices, flies infestations, and regular consumption of street food are also some determinant factors for the occurrence of diarrheal disease [7, 8]. The incidence of diarrhea is higher in the second half of the infant's life when inborn immunity is weak and exposure to contaminated weaning foods increases [3, 9].

Suboptimal infant and young child feeding practices are a major contributor to child morbidity and mortality [1, 5, 10]. The failure to exclusively breastfeed young infants to 6 months and the introduction of liquids and solid foods at the early age of life increase the risk of diarrheal disease and are an important cause of infant and young child morbidity and mortality in Africa. To reduce the diarrheal morbidity and mortality, the clinical and public health practitioner communities must work closely together to identify optimal diagnostic, treatment, and prevention methods. A better understanding of the interaction between persistent diarrhea and malnutrition as causes of mortality has drawn increased attention to the need to expand the scope of intervention programs [3, 5, 9, 11]. Oral rehydration solutions (ORS) or commercially available solutions made of appropriate amounts of sodium, potassium, and glucose should be used for rehydration if patients can consume or drink the required volumes; otherwise appropriate intravenous fluids may be used [10, 12]. Some studies and scholars recommended two general methods for an effective treatment of diarrheal disease, ORS and zinc supplementations [10].

In Ethiopia studies have shown that the prevalence of diarrhea both in urban and in rural areas was 13%. The prevalence of diarrhea was relatively high among children aged from 6 to 23 months. In southern Ethiopia, 25.5% of children experience diarrhea, but three-fourths occurred among rural children [2]. However, there is no study which documented the magnitude and factors associated with diarrheal disease in the rural communities of Arba Minch Zuria. Therefore, this study aimed to assess the prevalence of diarrhea and associated factors among under-five children in Arba Minch Zuria.

## 2. Methods

This cross-sectional community based study was carried out from January to February (dry season) 2012 in Arba Minch Zuria, Southern Ethiopia. Arba Minch Zuria is one of the 77 woredas (districts) in the Southern Nations, Nationalities and Peoples' Region of Ethiopia. In 2007 Central Statistical Agency reported that this district has 165,680 total populations of which 82,751 were males and 82,929 were females [11]. Most of the people depend on traditional subsistence agriculture. Fruit and vegetables were the two main agricultural products of Arba Minch Zuria [2, 11].

Multistage sampling technique was used to select households. From 29 rural kebeles (small villages), 9 kebeles were randomly selected. Then, 590 mothers of index children aged under five years were selected from reference populations who were obtained from the survey conducted before the main study was commenced. The systematic random sampling technique was used to take the mothers-child pairs from each of the selected kebeles (small villages). In case of more than one child in a given household, the lottery method was used to select single child.

The sample size was calculated using a formula for estimation of a single proportion as follows:

(1) $$\begin{array}{c} n=\frac{{\left(Z\alpha /2\right)}^{2}p\left(1-p\right)}{d^{2}\;}, \end{array}$$

where *Z* = standard normal variable at 95% confidence level (1.96); *P* = 25.5 (proportion about prevalence of diarhea) [2]; *d* = 0.05 (5% margin of error); and total sample size = 295.

Considering the design effect, total sample size was multiplied by 2. So, the final total sample size was 590. The study included mothers who had children aged less than 5 years and are permanent residents of selected kebeles (small villages). Mothers who come to visit their parents were not included.

Data were collected using WHO core questionnaires [13]. The survey included questions concerning sociodemographics, maternal and child characteristics, child feeding practices, and environmental health conditions. Data collectors were selected from local people depending on their ability to speak the local language and training was given to them. The questionnaire was pretested for its understandability by selecting some subjects before the data collections begin. Based on a pretest additional adjustment was made in terminologies and formatting of the questionnaire. Principal investigators and trained supervisors monitored the overall quality of the data collection.

In this study, diarrhea was defined as a child with loose or watery stool for three or more times during a 24-hour period in the household within two-week period prior to the survey as reported by the mother/caretaker of the child. Prevalence of diarrhea was defined as the total number of diarrhea cases divided by the total number of under-five children in the study in the given time interval.

Water source was defined as improved-water sources if the sources were either of the following: household connections, public standpipes, protected dug wells, and protected springs. Unimproved-water sources included unprotected dug wells, unprotected springs and rivers.

Proper waste disposal was defined as a way of disposing refuses which included burning, burying in a pit or storing in a container, and disposing in the designed site whereas disposing in open fields was considered as an unimproved-disposal method.

Home based water treatment was defined as methods employed for the purposes of treating water in the home using any of the following affordable water treatment techniques like boiling, filtration, and chlorination (chlorine).

Maternal knowledge about diarrhea was defined as knowledge of child caregivers, how diarrhea can be transmitted, and methods controlling diarrheal episodes. Information on maternal knowledge was assessed using a questionnaire consisting of questions on identification of diarrhea, seeking medical attention, treatment practices including amount and type of foods given, breastfeeding behavior during illness, prevention of diarrhea, and food handling practices.

Hand washing was defined as if a mother/caretaker had a practice of hand washing after toilet, before child fed, and before having contact with food or any contaminated utensils using clean water and soap or ash which was considered as “good” if not “poor.”

The data were entered in double EpiData software, checked for missing values and outliers and analyzed using SPSS (SPSS Inc. version 16.0, Chicago, Illinois). Descriptive statistics were used to describe the study population in relation to relevant variables. To identify independent predictors of childhood diarrhea the negative binomial regression model was used to control the effect of confounders. The test was two-sided and *P* < 0.05 was considered statistically significant. We reported the results as an adjusted prevalence ratio (APR) and 95% confidence intervals.

The study was conducted after getting official permission from an ethical clearance committee of Arba Minch University, College of Medicine and Health Sciences. Informed verbal consent was obtained from each study participant. Each respondent was informed about the objective of the study and privacy during the interview. Participants were informed about ways of prevention and home management of childhood diarrhea after collection of data. ORS was given to the mothers whose child had diarrhea during data collection time with clear instructions. Besides, they were also advised when to take the child to the nearest health facility so as to get professional support.

## 3. Results

A total of 590 households were included in this study and a complete response was obtained from all (100%) respondents. The mean age of mothers was 29.5 (SD ± 6. 7), the range being from 15 to 45 years. The majority of mothers 565 (95.7%) were married. More than two-thirds (66.90%) of households had a family size greater than 5 people. The majority of mothers 366 (62%) did not attend formal education (Table 1).

With regard to the sanitary facilities, 540 (91.5%) of households had latrine and 391 (62.27%) households got the water from protected spring and piped water. Almost all households 580 (98%) were living in a house made of mud floor. Majority of dwelling houses 390 (66%) had no partition room. Fifty (9%) of the households had no latrine. The mean per capita water consumption of the households was 6.5 (SD ± 4) litres/day. More than one-third of households (33.7%) used drinking water from unprotected sources. Only 191 (32.4%) mothers have a comprehensive knowledge about the cause of diarrhea and ways of transmission (Table 2).

Two-hundred forty-five children (41.5%) were aged 24 months and above. Among children who were currently breastfeeding, 131 (65.5%) were exclusively breastfeeding. Findings from this study showed that 180 (30.5%) children have experienced diarrhea in the last two weeks preceding the survey. The findings of the study also showed that, out of 180 mothers whose child had gotten diarrhea, about 31% of mothers did not give anything to manage the diarrhea (Table 3).

Diarrheal disease was significantly associated with maternal education, child age, and maternal hand washing practices. Multiple regression model showed that children whose mothers did not attend any formal education were 89% more likely to develop diarrhea (APR = 1.89 [95% CI: 1.35, 2.53]) compared to children whose mothers attended formal education (Table 4).

The age of children was also significantly associated with the occurrence of diarrhea. Children aged from 6 to 23 months were about 3 times more likely to develop diarrhea (APR = 2.78 [95% CI: 1.72, 4.55]) compared to children whose age was less than five months.

The risk of developing diarrhea was about 2 times higher among children whose mothers had poor hand washing practice (APR = 2.33 [95% CI: 1.80, 4.15]) compared to children whose mothers had good hand washing practice.

## 4. Discussion

Findings from this study showed that the prevalence of childhood diarrhea among under-five children was about 30.5%, which is relatively high when compared with 13% reported by Ethiopian Demographic and Health Survey in 2011 [2, 5]. That might be due to the inclusion of only rural children and the difference in provision health service between rural and urban population.

This study indicated that maternal education status had a significant contribution to preventing diarrheal morbidity. Children whose mothers did not attend formal education were more likely to develop diarrhea compared to children whose mothers did not attend any formal education. Findings from Ghana and Nigeria also showed that the prevalence of diarrhea varies according to education status of children's mothers, which is relatively high among children whose mothers had no education [8, 14, 15]. This might be due to health education given on hygiene, feeding and weaning practices and the management of childhood illness at school, and the role of different school clubs in personal hygiene and environmental sanitation.

This study showed that availability of latrine, types of floor, waste disposal system, and the source of water were not significantly associated with diarrheal morbidity an inverse of studies from Ghana, where availability of water, sanitary facilities, and hygienic practices were positively associated with the occurrence of diarrheal disease [2, 8]. This might be due to differences in culture of waste management and seasonal variation.

The children aged from 6 to 23 months were at high risk of developing diarrhea compared to children aged less than five months. The findings from Thailand [16] also showed that children whose ages from 6 to 23 months were more endanger of diarrheal disease than other age groups. This might be due to transition from an exclusive breastfeeding to introduction of complementary food and poor maternal hand washing and an increased introduction of solid foods which is unsafe and poor in hygiene to children whose immunity was not well developed.

This study also indicated that poor maternal hand washing practices were positively associated with the occurrence of diarrheal morbidity. This indicates that since the mothers were the main caregivers for their children they should wash their hands before feeding infants and young children to tackle the occurrence of hygiene related disease. Studies have also indicated that the proper hand washing before feeding this vulnerable age group has great role in the prevention of diarrhea and other diseases [2, 15, 17].

A large number of mothers, whose child had diarrhea, did not do anything to manage the diarrhea at home level. For the better management of diarrhea the national guideline recommends counseling mothers on the three rules of home treatments: give extra fluid, continue feeding, and advise the mother when to return to health facility [18]. Therefore, the health planners and health practitioners should work hard to bring behavioral change among the community for better perfection and management of diarrhea.

The findings of this study can be used as baseline data for policy makers in formulation, national infants and young health guideline and achievement of millennium development goals in the reduction of child mortality. Using WHO core questionnaire and community based study design is the strength of this study. However, the cross-sectional nature of this study limited drawing causal inferences from the association between the factors and the occurrence of diarrhea. There might be also reporting bias during face to face interviews which is tried to be minimized by selecting data collectors from the native local language speakers. There might be also recall bias, but we tried to limit recall bias by checking the card and the records about the child by health extension workers. Since this study was collected during dry season it might be difficult to consider the impacts of seasonal variation.

## 5. Conclusions

The findings of this study showed that the prevalence of diarrhea is high (30.5%). The occurrence of diarrhea was positively associated with maternal education, age of the child, and personal hygiene. Therefore, the findings in this study have important policy implications for health intervention and support the view that investing in girls' education may have substantial benefits for child health. Women's education level of at least primary should be achieved to reduce childhood diarrhea. Reducing diarrhea involves providing better sanitation for the entire population and the hygiene of the person caring for the child through community health workers. Although prevention is better than cure, sometimes prevention activities might fall and lead to morbidity. Therefore, counseling mothers on the three rules of home treatment (give extra fluid, continue feeding, and advise the mother when to return to health facility) is very crucial for the control and the prevention of the disease. We recommend further study to identify factors affecting home management of childhood diarrhea.

## Figures and Tables

**Table 1:** Socioeconomic characteristics of the households in Arba Minch Zuria, 2012.

Variables	Frequency	Percent (%)
Marital status of mothers		
Married	565	95.7
Single	25	4.3
Mothers' educational status		
No formal education	366	62.0
Primary school and above	224	38.0
Occupation of mother		
Housewife	517	87.7
Merchant	41	6.9
Others^*^	32	5.4
Fathers' educational status		
No formal education	357	60.5
Primary and above	233	39.5
Paternal occupation		
Farmers	517	87.60
Merchant	40	6.80
Daily laborer	15	2.50
Others	18	3.05
Ethnicity		
Gamo	462	78.71
Wolayita	91	15.50
Others^**^	34	5.79
Number of U5		
1	395	66.9
>1	195	29.1
Family size		
<5	195	29.1
≥5	395	66.9
Maternal age (in years)		
>20	12	2.1
20–24	117	19.80
25–29	186	31.5
30–34	125	21.5
≥35	150	25.42
Attended antenatal care		
Yes	446	75.6
No	144	24.4
Religion		
Protestant	371	62.9
Orthodox	205	34.75
Muslim	14	2.35

^*^Government employee; daily laborer; ^**^Amara, Gurage, and Oromo.

**Table 2:** The characteristics of children's living environment in Arba Minch Zuria, 2012.

Variables	Frequency (*n*)	Percent (%)
Floor of the house		
Mud	439	74.4
Cement	151	25.6
Number of rooms in the house		
1	390	66
2	110	18.6
≥3	90	15.4
Availability of latrine		
Yes	540	91.6
No	50	8.4
Type of latrine		
Traditional pit latrine	438	81.1
Ventilated improved latrine	102	18.9
Waste disposal system		
Proper disposal	475	80.5
Open field disposal	115	19.5
Source of drinking water		
Protected source	391	66.3
Unprotected source	199	33.7
Distance of water source		
≤30 minutes	503	85.4
>30 minutes	87	14.6
Ways of water transportation		
Covered container	496	84.1
Uncovered container	94	15.9
Home based water treatment		
Yes	93	15.7
No	491	84.3
Knowledge about cause of diarrhea		
Have knowledge	191	32.4
Have no knowledge	399	67.6

**Table 3:** Child demographic and behavioral characteristics in Arba Minch Zuria, 2012.

Variables	Frequency (*n*)	Percent (%)
Child sex		
Male	265	44.9
Female	325	55.1
Child's age		
0–5 months	193	32.7
6–23 months	152	25.8
24 months and above	245	41.5
Birth order		
First	94	16.1
Second	128	21.6
Third	129	21.8
Fourth and above	239	40.5
Exclusive breast feeding		
Yes	131	65.5
No	69	34.5
Measles vaccination		
Yes	56	53.3
No	49	46.7
Hand washing practice		
Good	508	86.1
Poor	82	13.9
Diarrhea		
Yes	180	30.5
No	410	69.5
Home management of diarrhea		
Yes	56	31.11
No	124	68.89
Child feeding methods		
Cup	155	26.30
Spoon	100	16.95
Hand	180	30.51
Bottle	25	4.24
Not any instruments	130	22

**Table 4:** Factors associated with diarrheal disease among under-five children in Arba Minch Zuria District, Southern Ethiopia, 2012.

Predictors	Diarrhea		CPR (95% CI)	APR (95% CI)
	Yes	No		
Mother's education				
No formal education	125 (34.2%)	241 (65.8%)	1.39 (1.06, 1.82)^*^	1.89 (1.35, 2.53)^*^
Primary school and above	55 (24.6%)	169 (75.4%)	1.00	1.00
Floor of the house				
Mud	145 (33%)	294 (67%)	1.42 (1.04, 1.96)^*^	1.22 (0.73, 2.04)
Cement	35 (23.2%)	116 (76.8%)	1.00	1.00
Knowledge about diarrhea				
Had knowledge	110 (27.6%)	289 (72.4%)	0.75 (0.59, 0.96)^*^	0.71 (0.45, 1.12)
Had no knowledge	70 (36.6%)	121 (63.4%)	1.00	1.00
Latrine available				
No	15 (30%)	35 (70%)	0.97 (0.63, 1.53)	—
Yes	165 (30.5%)	375 (69.5%)	1.00	
Waste disposal system				
Improper disposal	39 (33.9%)	76 (66.1%)	1.14 (0.85, 1.53)	—
Proper disposal	141 (29.7)	334 (70.3)	1.00	
Source of drinking water				
Unprotected source	66 (33.2%)	133 (66.8%)	1.14 (1.05, 1.49)^*^	1.32 (0.97, 1.72)
Protected source	114 (29.2)	277 (70.8%)	1.00	1.00
Age of the child				
0–5 months	45 (23.3%)	148 (76.7%)	1.00	1.00
6–23 months	60 (39.5%)	92 (60.5%)	1.69 (1.23, 3.34)^*^	2.78 (1.72, 4.55)^*^
24 months and above	75 (30.6%)	170 (69.4%)	1.31 (0.96, 1.80)	1.52 (0.86, 2.70)
Hand washing practice				
Poor	44 (54.4%)	38 (45.6%)	1.83 (1.44, 2.33)^*^	2.33 (1.80, 4.15)^*^
Good	149 (28.8%)	359 (71.2%)	1.00	1.00

^*^Statistical significance at *P* < 0.05, CPR: crude prevalence ratio, APR: adjusted prevalence ratio.

## References

[1] World Health Organization (WHO) (2010). *Integrated Management of Childhood Illness*.

[2] Central Statistical Authority (2011). *Ethiopia Demographic and Health Survey 2011*.

[3] Fischer Walker C. L., Rudan I., Liu L. (2013). Global burden of childhood pneumonia and diarrhoea. *The Lancet*.

[4] Gerald T., Keusch O., Alok B. (2001). *Disease Control Priorities in Developing Countries*.

[5] Federal Ministry of Health (2005). *National Strategy for Child Survival in Ethiopia*.

[6] World Health Organization (2000). *WHO World Water Day Report*.

[7] Yassin K. (2000). Morbidity and risk factors and diarrheal diseases among under-five children in rural Upper Egypt. *Journal of Tropical Pediatrics*.

[8] Oadi K., Kuitunen M. (2005). Childhood diarrheal morbidity in the Accra Metropolitan Area, Ghana: socio-economic, environmental and behavioral risk determinants. *Journal of Health and Population in Developing Countries*.

[9] Yohannes A. G., Streatfield K., Bost L. (1992). Child morbidity patterns in Ethiopia. *Journal of Biosocial Science*.

[10] USAID (2005). *Guidelines for New Diarrhea Treatment Protocols for Community-Based Healthcare Workers*.

[11] Central Statistical Agency (2007). Summary of statistical report of 2007. *Population and Housing Census*.

[12] Center for Disease Control (2008). *Guidelines for the Management of Acute Diarrhea*.

[13] WHO/UNICEF (2006). *Core Questions on Drinking Water and Sanitation for Household Surveys*.

[14] Yilgwan C. S., Okolo S. N. (2012). Prevalence of diarrhea disease and risk factors in Jos University Teaching Hospital, Nigeria. *Annals of African Medicine*.

[15] Yilgwan C. S., Yilgwan G., Abok I. I. (2005). Domestic water sourcing and the risk of diarrhea: a cross-sectional survey of a semi-urban community in Nigeria. *Journal of Medicine*.

[16] Calistus W., Alessio P. (2009). Factors associated with diarrhea among children less than 5 years old in Thailand: a secondary analysis of Thailand multiple indicator cluster survey. *Journal of Health Research*.

[17] Vieira G. O., Silva L. R., Vieira T. D. O. (2003). Child feeding and diarrhea morbidity. *Jornal de Pediatria*.

[18] Drug Administration and Control Authority of Ethiopia Contents (2010). *Standard Treatment Guideline for Primary Hospitals*.

